# A high-selenium lentil dietary intervention in Bangladesh to counteract arsenic toxicity: study protocol for a randomized controlled trial

**DOI:** 10.1186/s13063-016-1344-y

**Published:** 2016-04-27

**Authors:** Regina M. Krohn, Rubhana Raqib, Evana Akhtar, Albert Vandenberg, Judit E. G. Smits

**Affiliations:** Department of Ecosystem & Public Health, Faculty of Veterinary Medicine, University of Calgary, 3280 Hospital Drive, Calgary, AB T2N 4Z6 Canada; International Centre for Diarrhoeal Disease Research, Bangladesh (icddr,b), 68, Shaheed Tajuddin Ahmed Sarani, Mohakhali, Dhaka, 1212 Bangladesh; Department of Plant Sciences, University of Saskatchewan, 51 Campus Drive, Saskatoon, SK S7N 5A8 Canada

**Keywords:** Chronic arsenic poisoning, High-selenium lentils, Arsenic body burden, Dietary intervention

## Abstract

**Background:**

Millions of people worldwide are exposed to dangerous levels of arsenic (above the WHO water standard of 10 ppb) in drinking water and food. Lack of nutritious foods exacerbates the adverse health effects of arsenic poisoning. The micronutrient selenium is a known antagonist to arsenic, promoting the excretion of arsenic from the body. Studies are in progress examining the potential of using selenium supplement pills to counteract arsenic toxicity. We are planning a clinical trial to test whether high-selenium lentils, as a whole food solution, can improve the health of arsenic-exposed Bangladeshi villagers.

**Methods/design:**

A total of 400 participants (about 80 families) will be divided into two groups via computer-generated block randomization. Eligibility criteria are age (≥14) years) and arsenic concentration in the household tube well (≥100 ppb).

In this double-blind study, one group will eat high-selenium lentils grown in western Canada; the other will consume low-selenium lentils grown in Idaho, USA. Each participant will consume 65 g of lentils each day for 6 months. At the onset, midterm, and end of the trial, blood, urine and stool, plus hair (day 1 and at 6 months only) samples will be collected and a health examination conducted including assessment of acute lung inflammation, body mass and height, and blood pressure. The major outcome will be arsenic excretion in urine and feces, as well as arsenic deposition in hair and morbidity outcomes as assessed by a biweekly questionnaire. Secondary outcomes include antioxidant status, lipid profile, lung inflammation status, and blood pressure.

**Discussion:**

Selenium pills as a treatment for arsenic exposure are costly and inconvenient, whereas a whole food approach to lower the toxic burden of arsenic may be a practical remedy for Bangladeshi people while efforts to provide safe drinking water are continuing. If high-selenium lentils prove to be effective in counteracting arsenic toxicity, agronomic partnerships between Canada and Bangladesh will work to improve the selenium content of the Bangladeshi-grown lentil crops. Results will be presented to the community to promote informed food choices, which may include increasing selenium in their diet.

**Trial registration:**

ClinicalTrials.gov NCT02429921

**Electronic supplementary material:**

The online version of this article (doi:10.1186/s13063-016-1344-y) contains supplementary material, which is available to authorized users.

## Background

In the 1970s, the United Nations International Children’s Emergency Fund (UNICEF) and the Bangladeshi government, followed by private initiatives, installed millions of tube wells in rural Bangladesh in an effort to reduce infant and child mortality caused by water-borne diseases such as cholera and other diarrheas [1, 2]. Tragically, many of these deeper wells contained arsenic, which leaches from the bedrock into the groundwater. It was not until the 1990s, when people developed previously unknown skin lesions, that more extensive well water testing revealed the scale and severity of the arsenic problem. By then millions of people had been exposed to arsenic for more than 20 years [3].

In 2002, the World Health Organization (WHO) described the case of Bangladesh as “the world’s largest mass poisoning of a population in history…” [4]. At that time it was estimated that between 35 and 77 million people were exposed to dangerously high concentrations of arsenic — above the WHO guideline of 10 ppb and frequently above the national standard in Bangladesh of 50 ppb. It is noteworthy that food such as rice is irrigated with arsenic-contaminated shallow tube well water, potentially adding to the toxic burden [5].

The annual cost to treat arsenic-related health problems in Bangladesh is currently estimated at up to $77.5 million [6].

A number of studies have found an association between the detrimental effects of arsenic exposure and malnutrition [7–9], which is also a prominent health concern in the Bangladeshi population, especially in children [10]. Chen et al*.* found that the risk of developing arsenic-related skin lesions was greatly enhanced with low blood selenium. Selenium and arsenic work antagonistically in the body by competing in many biological functions [11]. In the blood, selenium interacts with arsenic to form a complex which is excreted in the bile [12], thereby lowering the arsenic body burden. Thus, higher selenium intake may be crucial to combat arsenic toxicity. Selenium levels in food are dependent on selenium bioavailability in the soil. Limited data are available on the selenium content of soils in Bangladesh. Initially it was found that agricultural soil in Bangladesh (*n* = 19) contains good selenium levels [13]. However, the authors determined that only 12 % of selenium was soluble, reducing the immediately available selenium to 0.12 μg/g. A ”nutritional selenium deficiency” in soil is < 0.5 μg/g soil [14]; therefore, Bangladeshi soil falls into the selenium deficient category. The US dietary recommended daily allowance (US-RDA) for selenium for an adult is 55 μg/day [15], and Spallholz et al*.* (2008) estimated that Bangladeshis consume about 26 μg/day [13], approximately half of the RDA.

Supplementation of selenium in pill form is currently recommended to treat arsenicosis. However, pills are not often well received by people and are expensive for low-income families. Lentils are a very common food in Bangladesh, with the major lentil imports coming from Canada, a world leader in lentil exports. In crop year 2011/2012, 47,620 tonnes (metric tons) of lentils were exported to Bangladesh, and in 2012/2013 this increased to 90,800 tonnes (metric tons) [16]. Saskatchewan (SK) grown lentils are naturally rich in selenium, mostly in the form of L-selenomethionine, which is highly bioavailable [17]. Saskatchewan lentils have mean selenium levels between 425–672 μg/kg, with the levels in some regions as high as 1884 μg/kg [18]. Experimental studies in animal models demonstrated that diets made of high-selenium lentils counteract arsenic toxicity and reduce arsenic-related atherosclerosis [19, 20]. Incorporated into the daily meals of arsenic-exposed Bangladeshi families, high-selenium lentils may help mitigate the symptoms of long-term arsenic poisoning in a cost-effective, culturally acceptable, and nutritionally beneficial way.

### Trial objectives

The hypothesis of the trial is that consumption of high-selenium lentils from Saskatchewan, Canada, will reduce the body burden of arsenic and decrease arsenic toxicity in a chronically arsenic-exposed Bangladeshi population compared to those consuming low-selenium lentils.

The primary objective was to perform a double-blind, randomized controlled trial to determine whether incorporation of high-selenium lentils into the daily diet, providing at least the RDA of Se (55 ug), can decrease the body burden of arsenic, evident by higher excretion in urine and/or feces and decreased deposition in hair, compared to the treatment group on low-selenium lentil consumption. Secondary objectives were to determine if consumption of high-selenium lentils:

1. improves antioxidant status
2. reduces oxidative DNA damage
3. changes acute phase proteins as well as serum lipid levels in arsenic-exposed individuals
4. decreases arsenic-related lung inflammation measured using exhaled nitric oxide as an indirect indicator
5. improves the body mass index (BMI) and blood pressure of arsenic-exposed individuals

Herein, we describe the design and methods protocol for our 3-year study now underway.

## Methods/design

This trial protocol has been approved by the University of Calgary’s Conjoint Health Research Ethics Board (Ethics ID: REB13-1211-Mod3), the University of Saskatchewan’s Biomedical Research Ethics Board (Bio-No. 14-212), plus the Research Review Committee, the Ethics Review Committee, and the Data and Safety Monitoring Board (DSMB) of the International Centre for Diarrhoeal Disease Research, Bangladesh (Icddr,b) (Protocol No. PR-14013). Any changes to the trial protocol are communicated to the relevant parties and ethics boards. The trial will be reported according to SPIRIT (Standard Protocol Items: Recommendations for Intervention Trials) 2013 [21] (see Additional file 1). A summary of the trial according to the WHO Trial Registration Minimal Data Set is provided in Table 1 [22].

**Table 1:** WHO Trial Registration Minimal Data Set according to Moja et al*.* [22]

Data	Information
Trial identification number	ClinicalTrials.gov NCT02429921
Trial registration date	April 21, 2015
Secondary IDs	REB13-1211 (CHREB, University of Calgary), PR-14013 (ERC, icddr,b)
Funding sources	Global Institute for Food Security (GIFS); Grand Challenges Canada - Stars in Global Health Round 5
Primary sponsor	JEGS, Faculty of Vet. Med, University of Calgary, Canada
Secondary sponsor	RR, icddr,b, Dhaka, Bangladesh
Responsible contact person	RR, icddr,b; rubhana@icddrb.org
Research contact person	JEGS, Faculty of Vet. Med., University of Calgary; jegsmits@ucalgary.ca
Brief title of the study	High-selenium Lentils Versus Arsenic Toxicity
Official title of the study	Mitigating arsenic toxicity in Bangladeshi people by supplementing their diets with high selenium lentils
Country of recruitment	Bangladesh
Health problem studied	Chronic arsenic poisoning
Intervention	High-selenium lentils, control: low-selenium lentils
Key inclusion and exclusion criteria	Inclusion criteria: families with >100 ppb As in their well water; ages eligible for study: 14–75
	Exclusion criteria: As content of well water ≤100 ppb; ill health detected during initial recruitment health check
Study type	Double blind, parallel, block-randomized, controlled intervention trial
Trial start date	September 28, 2015
Target sample size	200 per treatment group (400 in total)
Recruitment status	Active
Primary outcome	Arsenic body burden: We will determine if high-Se lentil consumption will decrease the body burden of As by measurement of As excretion in urine and feces and deposition in hair
Key secondary outcomes	We will determine if high-Se lentils:
	1. Increase concentrations of protective antioxidants and reduce oxidative stress and acute phase response in blood
	2. Modulate serum lipid profile and blood pressure
	3. Reduce As-related lung inflammation

### Study settings

The study is a parallel, double-blind, randomized controlled trial. Participants for this trial will be recruited from the communities of Shahrasti and Chandpur, Bangladesh, where arsenic levels in tube well water frequently exceed the national standard of 50 ppb. Families in these rural areas typically have their own tube well in the yard from which they collect their drinking and cooking water. Entire families will be recruited.

### Inclusion criteria

The inclusion criteria are as follows:

1. Families who are exposed to arsenic levels ≥100 ppb in their drinking water will be included in the trial.
2. All family members aged 14 and above will be enrolled if they agree.

### Exclusion criteria

The exclusion criteria are as follows:

1. Children under the age of 14 will not be included in the trial, although they will also eat the high-selenium or control lentils like the rest of the family.
2. Families whose tube well water has arsenic levels <100 ppb will be excluded from the trial.
3. If any family member(s) appears unhealthy, anemic for example, the family will not be recruited.

#### Justification for eligibility criteria

The national standard for arsenic in drinking water is 50 ppb. To increase the possibility of detecting clinical differences between the two treatments, we chose families with wells having arsenic levels above the national standard and ≥100 ppb. Moreover, after testing all the tube wells of potential participants, we identified only 10 out of 118 wells that had arsenic levels between 50 and 100 ppb. This resulted in a small sample size for this subgroup, which may not produce interpretable results and could weaken the statistical power of the whole trial.

We planned to include children of ages 10–17 in the trial, as they are a vulnerable group and actively developing, especially those from ages 10 to 14. The observation of changes in nutritional status in these children due to lentil consumption would be especially valuable in our opinion. However, the Ethics Review Committee at icddr,b stipulated that children under the age of 14 be excluded from the study. Nevertheless, all family members of study participants, including young children, will eat the high-selenium or control lentils.

### Consent

Informed consent will be obtained from all adults, which includes their children in the trial, and assent forms are collected from every recruited child between 14–18 years of age (see Additional files 2 and 3). All forms have been translated into Bangla, and the recruiters will explain the trial procedures in detail and answer any questions the participants may have.

### Interventions and compliance

Families will be randomly assigned to one of two interventions:

1. High-selenium lentils
2. Low-selenium lentils

For a period of 6 months, each participant will be required to eat 65 g lentils each day. The group that receives the high-selenium lentils will therefore consume at least the US-RDA of 55 μg selenium per day. Apart from lentil supply, the other food consumed daily will not be controlled, so study participants who are in the low-selenium lentil group will also receive selenium from other food sources as usual, but this is expected to be similar between groups. The female family head will measure 65 g lentils per family member each day (a measuring cup will be provided) and will prepare foods, such as dal (lentil soup) and lentil patties, that are generally eaten by the family members over the day. The field staff will visit families twice a week in the first month of the intervention to promote and record compliance in lentil consumption. To ensure and confirm compliance, the lentil supply will be distributed weekly, and study participants will complete a lentil consumption questionnaire on a weekly basis for the duration of the trial.

We performed a 1-week pilot trial with four families to determine likelihood of compliance. The four families received the adequate amount of lentils (purchased from the local market) for 1 week, and consumption was monitored. All families did eat the required amount of lentils. The female family head used different recipes to incorporate the lentils into the daily meals.

### Safety considerations

We do not expect any adverse effects from lentil consumption regardless of the treatment groups since both types of lentils are already traded and consumed worldwide. The group that will consume the high-selenium lentils will receive the US-RDA of 55 μg of selenium per day, plus the average of 23 μg selenium in the regular Bangladeshi diet acquired through other components, primarily fish and meat [13]. The current tolerable upper intake levels of selenium for humans are 280 μg/day (9–13 years) and 400 μg/day (ages 14+) according to the Dietary Supplement Fact Sheet from the National Institutes of Health, USA [23]. Supplementation of selenium at 200 μg/day in studies of up to 13 years duration — in addition to other dietary selenium intake — lowers the risk of cancers, enhances cell-mediated immune function, decreases progression of chronic infectious diseases, inflammation and autoimmune diseases, and presents no evidence of toxicity [24–27]. Hence, even if study participants consumed double the amount of lentils a day, their selenium intake would be well below toxic levels and would likely be beneficial to their health.

An independent DSMB at icddr,b has been formed which will conduct periodic meetings and report the occurrence of adverse or seriously adverse events, if any. In this highly unlikely event, unblinding would be permissible.

### Sample size

We determined the sample size for this trial with an online power and sample size calculator (www.powerandsamplesize.com), based on a one-sided effect for arsenic concentration in hair.

Based on a previous intervention trial [28], in which arsenic-exposed participants received selenium-enriched yeast (100–200 μg/day, an amount two to four times higher than our dietary treatments), we calculated the sample size from their data generated after a 9-month intervention. Using the mean arsenic levels in hair of the control and selenium-treatment groups, Mean_A_ = 2.01 μg/g (±1.4) and Mean_B_ = 1.23 (±0.73), respectively, and a type I error of 0.05 and a power of 0.80, we calculated a minimum participant number of 26 per treatment group. This sample size underestimates our needed sample size due to substantial differences from our planned trial. Considering the up to fourfold lower selenium treatment in our trial, we felt it was prudent to quadruple the number (=100) and then double it to accommodate our larger age range, shorter duration, and loss to follow-up, arriving at the final participant number of 200.

### Randomization and blinding

This trial will be double-blinded. High- and low-selenium lentils have been packed in color-coded bags (green and white), and neither the study participants nor the study investigators know of the lentil type.

Related families commonly live in the same Bari (group of homes on one plot of land) in rural Bangladesh, with families, defined as members taking meals from the same cooking pot, living in individual homes or households (HHs) in the Bari. All family members in an HH will receive the same lentil type. However, within each Bari individual HHs may receive different lentil types. Once families are recruited, they will be assigned to treatment group A or B (deficient or high-selenium lentil groups) using a computer-generated block randomization method, and one group will be randomly assigned to receive selenium-rich lentils. The sample size is 200 individuals per group. Typically a family consists of four to eight members. Thus, if there are six members per HH, there will be approximately 34 HH per group. For the block randomization we may consider blocks of variable length, e.g., four or six HHs per block; thus, in each block there will be four or six HHs. Equal numbers of HHs are distributed in the two groups, and either group may receive the selenium-rich lentils. All HHs will receive their supply of lentils weekly. Sequential numbers will be allocated to the participants, e.g., 010101, 010102 (Bari 01, HH 01, family member 01, 02, 03…), etc.

### Lentil production and analysis

Small, red, fast-cooking lentils, the preferred kind in Bangladesh, were grown for the trial. Lentils grown in the northwestern USA are low in selenium [18]. In the summer of 2014 a location near Genesee, Idaho was chosen for growing the low-selenium lentils, while the high-selenium lentils were grown at Lucky Lake, Saskatchewan, Canada. All lentils were processed (dehulled, polished, and bagged in colored 25-kg bags) in a lentil dehulling and exporting facility in Vanscoy, Saskatchewan.

Samples of the lentils were analyzed for essential minerals, phytic acid, and macronutrients (Table 2). Idaho lentils contain 0.029 ppm selenium; the Saskatchewan lentils contain 0.854 ppm selenium. The lentils were tested for radiation biosafety certification by the Saskatchewan Research Council, Saskatoon, which declared the two lentil lots fit for human consumption.

**Table 2:** Macronutrient and micronutrient content of the two lentil types

Macronutrients	Measure	Sask. lentils	Idaho lentils
Protein	% by weight	26.22	27.73
Starch	% by weight	38.00	37.00
TDF	% by weight	8.48	6.66
Fat	% by weight	0.78	0.77
Ash	% by weight	2.77	3.16
Phytochemicals			
Phytic acid	g/kg	0.61	0.72
Minerals			
Calcium	mg/kg	327.88	377.51
Potassium	g/kg	10.45	10.94
Sodium	mg/kg	71.74	75.00
Magnesium	mg/kg	786.30	943.00
Copper	mg/kg	9.34	11.44
Iron	mg/kg	75.75	65.3
Zinc	mg/kg	42.15	51.9
Manganese	mg/kg	16.95	14.61
Selenium	mg/kg	0.854	0.029
Arsenic	mg/kg	<0.001	<0.001

Abbreviation: *TDF* total digestible fiber

### Lentil shipment and storage

Five metric tons of each lentil type were shipped from Vanscoy, Saskatchewan, to the port in Chittagong, Bangladesh, where they were tested again for radiation and phytotoxins, a protective measure to prevent importation of contaminated grain. Once released the lentils were trucked to icddr,b Dhaka, where each bag was uniquely numbered and stored at room temperature.

### Recruitment

At present, we are recruiting study participants. Two senior field site staff and three village health workers are visiting eligible HHs to obtain consent/assent from adults and children, respectively. The staff will collect sociodemographic data at that time via questionnaire.

### Study procedures

After obtaining consent, families (four to six per week) visit the field office for baseline sample collection and physical examination and for completing the initial morbidity questionnaire. After this first visit, weekly lentil distribution and consumption starts.

**Fig. 1:**
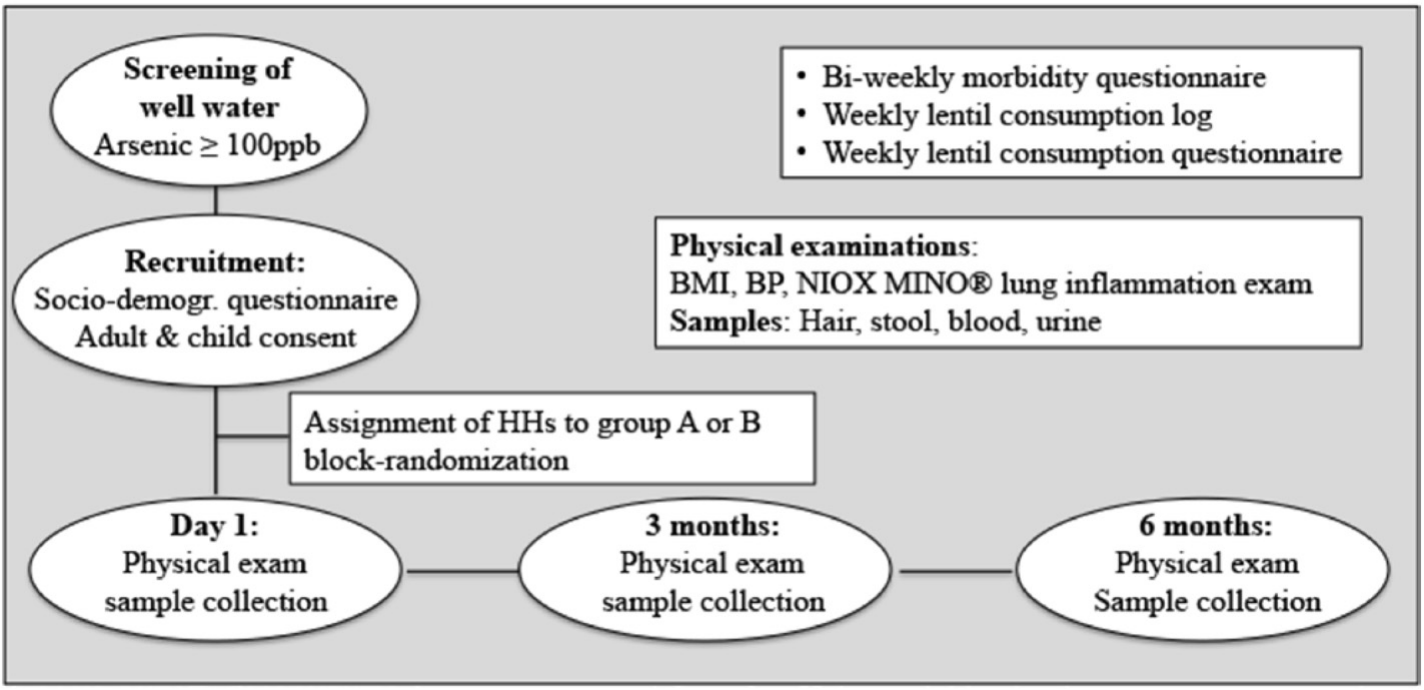
Flow chart of the dietary intervention trial; BMI, body mass index; BP, blood pressure; HHs, households

The flow chart shows the complete 6-month study procedures (Fig. 1).

At baseline (day 1), midterm (3 months), and at the end of the study (6 months), samples will be collected as described in the following paragraph.

Urine (15–20 ml) will be collected in trace-element free tubes, aliquoted, and stored at –80 °C. Stool samples (5–10 g) will be collected in trace-element free tubes from a subset (about 40) of participants and stored at –20 °C. Blood (10 ml/visit) will be collected in trace-element free tubes (S-Monovette®, Sarstedt). Blood samples, in cool boxes, will be sent to the Dhaka Lab on the same day for separation into plasma and cells, and aliquots of plasma will be stored at –80 °C. Buffy coat cells will be stored in an RNAlater freezer at –80 °C. Whole blood (100 μl) will be stored at –80 °C. Hair will be collected from the back of the head, close to the nape of the neck (approximately 0.2 g) for analysis of arsenic.

Samples may be stored for up to 5 years for future analyses.

The following physical examinations will be performed at baseline, midterm, and end of study:

1. Height and weight are measured to calculate body mass index (BMI).
2. Blood pressure (BP) is tested because of the association between arsenic and hypertension [29].
3. Lung inflammation (NIOX MINO®) — study participants exhale into a machine which detects exhaled nitric oxide, a marker for eosinophilic airway inflammation [30]. Previous reports showed an association of arsenic exposure with respiratory effects and inflammatory response of airway cells [31, 32].

### Laboratory methods

Selenium will be measured in blood by graphite furnace atomic absorption spectrometry (GFAAS). Arsenic in hair, stool, and urine samples will be measured by hydride generation atomic absorption spectrometry (HGAAS) after acid digestion of samples. For determining oxidative stress and antioxidant status, we will measure 8-hydroxy-2'-deoxyguanosine (8-OHdG), a major product of reactive oxygen species (ROS)-induced oxidative stress, in urine/plasma using commercial ELISA kits. Selenium-dependent glutathione peroxidase uses reduced glutathione (GSH) to scavenge ROS, resulting in the production of oxidized GSSG [33]. The antioxidant status (GSH/GSSG) will be assessed with ELISA analysis as described previously [20, 34]. Lipid profiles will be determined in serum by measuring triglyceride, total cholesterol, high-density lipoprotein (HDL), and low-density lipoprotein (LDL) cholesterol using commercial kits from Roche diagnostics on their automated clinical chemistry analyzer Cobas e601 (Roche Diagnostics, Mannheim, Germany). Acute phase response will be determined by analyzing Hs-CRP and α_1_-acid glycoprotein (AGP) in serum using the automated analyzer.

### Data analysis

Data are stored in a password-protected database at icddr,b, through a platform that allows sharing of data between project investigators (PIs) at icddr,b and the University of Calgary. Statistical analyses will be done using the statistical software SPSS for Windows PASW version 20. Normality and homogeneity of variances will be determined. Data not normally distributed will be transformed as necessary. Initially Spearman’s correlation analyses will evaluate univariate association between arsenic exposure (urinary and hair arsenic) and outcome variables (oxidative stress, antioxidant status, lung inflammatory status, and genetic variables). Covariates (e.g., demographic and socioeconomic variables, morbidity, daily diet, and BMI of participants) will be evaluated for significant association with exposure and outcomes. Missing data are excluded from that specific analysis. Significantly correlated variables will be further analyzed in multiple linear regression models controlling for potential confounding variables stratified by sex. Potential confounders will be identified based on correlations (*p* < 0.15) with the exposure (toxic metal) and outcomes and will be included in the final models when they change the effect estimate for metal exposure on the outcome by 5 % or more.

## Discussion

This trial is the first to use a high-selenium food source, i.e., lentils, in an attempt to combat the adverse health effects of chronic arsenic poisoning. Since lentils are an excellent source of non-animal protein and micronutrients such as Zn and Fe [35], we expect to see an improved BMI after 6 months of supplementation and possibly improved overall health status in all participants of the trial, regardless of the selenium content of the lentils. As a result, some of the proposed biomarkers may not be significantly different between the control and the high-selenium groups. However, since selenium can form a complex with arsenic, selenium supplementation, in principle, can reduce arsenic toxicity. We expect to see a significant decrease in the body burden of arsenic, the primary outcome of this study, in the high-selenium group. Mild beneficial effects of selenium have been indicated in a number of supplementation trials with selenium alone or in combination with other micronutrients [36–38]; more trials are needed for conclusive evidence.

If we can prove that health benefits occur due to consumption of high-selenium lentils in arsenic-exposed communities, the first step will be to inform the Bangladeshi public — through a follow-up meeting with the participants and local media — and to promote consumption of higher-selenium food sources. Here again, the female family head will be the one responsible for purchasing and preparing nutritious food for the family.

Furthermore, we will disseminate the study results to the Food Planning and Monitoring Unit (FPMU) of the Ministry of Food, the Government of Bangladesh, and provide evidence-based advice for policy formulation on lentil import and agricultural aspects. The results may guide Bangladeshi importers to specifically target importation of high-selenium lentils.

The long-term goal toward food security will entail working on agronomic techniques to increase the production of high-selenium lentils in Bangladesh.

## Trial status

Participants are currently being recruited.

## Additional files

Additional file 1: SPIRIT_checklist. (DOC 121 kb) Additional file 2: Adult consent form. (PDF 987 kb) Additional file 3: Child assent form. (PDF 88 kb)

## Abbreviations

8-OHdG: 8-hydroxy-2'-deoxyguanosine
BMI: body mass index
BP: blood pressure
CRP: C-reactive protein
GFAAS: graphite furnace atomic absorption spectrometry
GSH: reduced glutathione
GSSG: oxidized glutathione
HDL: high-density lipoprotein
HGAAS: hydride generation atomic absorption spectrometry
HH: household
LDL: low-density lipoprotein
ppb: parts per billion
RDA: recommended daily allowance
ROS: reactive oxygen species
