# Supplementary material for: A high-selenium lentil dietary intervention in Bangladesh to counteract arsenic toxicity: study protocol for a randomized controlled trial
Source: Trials. 2016 Apr 27;17:218. doi: 10.1186/s13063-016-1344-y (PMC4848822; doi:10.1186/s13063-016-1344-y)
Supplement: Additional file 2: — Adult consent form. (PDF 987 kb) [file 13063_2016_1344_MOESM2_ESM.pdf]

Judit E. G. Smits (DVM, MVetSc., PhD)
Professor, Wildlife & Environmental Toxicology
Department of Ecosystem & Public Health,
Faculty of Veterinary Medicine, Univ. of Calgary
3280 Hospital Drive, NW, Calgary, AB, Canada T2N 4N1
Email: jegsmits@ucalgary.ca

Adult Consent Form

TITLE: Mitigating arsenic related health problems in Bangladesh by introducing high-selenium lentils into the everyday diet

SPONSOR: Grand Challenges Canada, and the Global Institute for Food Security (GIFS)

INVESTIGATORS: Dr. Judit E.G. Smits, Faculty of Veterinary Medicine, Dept. of Ecosystem and Public Health; Co-I Dr. Rubhana Raqib, icddr,b, Dhaka, Bangladesh

Dr. Smits Phone: +1 403 210 7407
Dr. Raqib Phone: +8802-9827068

This consent form is only part of the process of informed consent. It should give you the basic idea of what the research is about and what your participation will involve. If you would like more detail about something mentioned here, or information not included here, please ask. Take the time to read this carefully and to understand any accompanying information. You will receive a copy of this form.

BACKGROUND

Arsenic poisoning from drinking water with high arsenic levels is a big health problem in Bangladesh, causing skin diseases, cancer and heart disease. In some areas in Bangladesh, wells that could provide water with low arsenic levels are not yet established due to the high costs and other complications involved. Therefore, other means of reducing the harmful effects of arsenic are necessary.
Selenium (Se) is a trace element in food that is essential for our health, and it can also counteract toxic effects of arsenic. Selenium also helps with the removal of arsenic from the body.

Judit E. G. Smits (DVM, MVetSc., PhD)
Professor, Wildlife & Environmental Toxicology
Department of Ecosystem & Public Health,
Faculty of Veterinary Medicine, Univ. of Calgary
3280 Hospital Drive, NW, Calgary, AB, Canada T2N 4N1
Email: jegsmits@ucalgary.ca

Bangladeshi soil has very low Se content, and thus lentils grown in Bangladesh are also low in Se. Lentils grown in Saskatchewan, Canada have a high Se content. We will enroll about 80 families (400 people) in your area, who are exposed to arsenic. They will be divided into 2 groups. One group will receive high-selenium lentils, the other low-selenium control lentils. The study will be blinded, that means neither the participants nor the research team, knows which lentil kind each family receives. But in the case of a medical emergency, where this information is needed, it will be disclosed to you and your treating physician.

WHAT IS THE PURPOSE OF THE STUDY?

The major aim of this study is to evaluate the health benefits of eating high-Se lentils by people who are exposed to arsenic in their drinking water.

WHAT WOULD I HAVE TO DO?

As one of the families living in an area with higher arsenic levels in drinking water, we ask you and your family members to participate in this 6 month trial. Should you agree to participate and allow your child/children to participate, we will ask the following of you:

1. The families will be expected to eat 65 grams of lentils per person in form of dahl throughout the day (a 65 g scoop will be provided for measurement). Your family will either receive high selenium lentils or low selenium lentils. Which kind of lentils your family will receive, will be left to chance (like the flip of a coin). Nobody in the study team will know whether you have been given the high selenium or the low selenium lentils.
2. Field assistants will visit you weekly to distribute the weekly lentil supply and fill in a questionnaire about how much of the lentil supply you ate.
3. Also, female village health workers (FVHW) will visit you twice a week to check if you are eating the lentils and talk about any questions you have.
4. Every 2 weeks, the FVHW will ask you questions about your health and take notes. This will take about 10 minutes.
5. You/your children will be interviewed/examined at 3 different times at the Field Office.

Judit E. G. Smits (DVM, MVetSc., PhD)
Professor, Wildlife & Environmental Toxicology
Department of Ecosystem & Public Health,
Faculty of Veterinary Medicine, Univ. of Calgary
3280 Hospital Drive, NW, Calgary, AB, Canada T2N 4N1
Email: jegsmits@ucalgary.ca

1) visit to Field Office, day 1: A baseline questionnaire will be given to gather information on your occupation, socioeconomic status, age, gender, etc. and habits such as smoking or chewing betel nut. To check for lung inflammation, you/your child will be asked to take a deep breath through a plastic mouthpiece attached to a small machine. When the lungs are full, you/your child will blow out through the same mouthpiece. The machine will analyse the breath.
   A health professional will measure your/your children's overall health and Body Mass Index (BMI) like height and weight, and blood pressure (BP) (BP only for adults).

   You/your children will be asked for following samples:
   10 ml blood (2 teaspoons) drawn from the arm with a sterile syringe; 10 ml urine; hair (approximately 1 x 1.5 cm patch cut from the back of the head at the nape of the neck, as close to the scalp as possible); and stool (about 4-5 g) will be obtained in the special containers given to your family.
2) visit in Field Office, at 3 months: Blood, urine, stool, lung inflammation, BMI, BP check as above.
3) visit in Field Office, at 6 months: Blood, urine, stool, hair, lung inflammation, BMI, BP check as above, plus another overall health assessment by a health professional.

6. Transportation to the Field Office will be provided and paid for by the research team. We will provide snacks at the Field Office. You may need to wait for up to 2 hours at the Field Office.

WHAT ARE THE RISKS?

Sterile disposable syringes and needles will be used for all the collection of blood. The insertion of the needle will cause a brief and temporary pain and there is a small risk of bruising. We will take all care to avoid any complications. For the lung inflammation test, some people may experience temporary shortness of breath (very rare), but it will get better in 1 or 2 minutes after the test.
Transportation to the field office with a rickshaw entails a minimal risk, but no more than in your everyday life.

Judit E. G. Smits (DVM, MVetSc., PhD)
Professor, Wildlife & Environmental Toxicology
Department of Ecosystem & Public Health,
Faculty of Veterinary Medicine, Univ. of Calgary
3280 Hospital Drive, NW, Calgary, AB, Canada T2N 4N1
Email: jegsmits@ucalgary.ca

You might have to wait at the field office. We will try to keep the waiting times short, and will provide snacks and drinks in the waiting room.
All families, whether receiving low-selenium or high-selenium lentils, will most likely benefit from the study, as the lentils they will eat are very nutritious, even if they have low selenium. Also, the results of this study are hoped to benefit society in general because it will help us to determine whether a simple solution like consumption of high-Se lentil will help to reduce arsenic-induced health problems in the community. This information may be useful for establishing agricultural and trade policies in the future.

If we find that you and your family are exposed to arsenic levels higher than the national standard of 0.05mg/L, we will let you know, and try to find a safer water source near you.

WILL I BENEFIT IF I TAKE PART?

If you agree to participate in this study there may or may not be a direct benefit to you. You are in the study because you are being exposed to arsenic in your drinking water, and your overall health status may improve over the period of the study, but it is unlikely that you will notice improvement in more serious arsenic-related ailments because this study is short-term, only 6 months, and the problems may have taken years to develop. Your overall health status may improve whether you receive the low-selenium or high-selenium lentils, as both kinds contain many other important, good nutrients regardless of selenium content.
Also, the results of this study are hoped to benefit society in general because it will help to understand whether a simple solution like eating high-Se lentil will help to reduce arsenic-induced toxicity in your community. This information may be useful for establishing agricultural and trade policies in the future.

Judit E. G. Smits (DVM, MVetSc., PhD)
Professor, Wildlife & Environmental Toxicology
Department of Ecosystem & Public Health,
Faculty of Veterinary Medicine, Univ. of Calgary
3280 Hospital Drive, NW, Calgary, AB, Canada T2N 4N1
Email: jegsmits@ucalgary.ca

DO I HAVE TO PARTICIPATE?

Your participation in this study is completely voluntary and confidential. At any time, you/your children may discontinue your/his/her participation in the study without any penalty or loss of benefit, but you must let us know by calling Dr. Raqib (number below) or telling one of the FVHW. If you decide to withdraw from the study, all data that you provided to the study will be destroyed, unless you give us permission to use the data and samples we already acquired from you. If some member(s) of the family decide to stop participating in the study, you may still continue to eat the lentils we provide. If the entire family withdraws from the study, we will not provide more lentils.
If new information becomes available that might affect your willingness to participate in the study, you will be informed as soon as possible.

WILL I BE PAID FOR PARTICIPATING, OR DO I HAVE TO PAY FOR ANYTHING?

You will not have any expenses related with this study. Transportation to the Field Office will be provided and paid for by the research team. As waiting times may be prolonged at the Field Office, drinks and snacks will be provided. You will not be paid for participating.

WILL MY RECORDS BE KEPT PRIVATE?

The principal investigators and research assistants that are involved with doing and evaluating the study will use the information collected.
The participating families and family members will have code names, or numbers generated by a computer program.

Only the researchers and the supervisors will be allowed to see any of the answers you give in the interview, and there will be no names on the interview protocol. Results from all participants will be just part of a group and presented as a summary. No individual information will be presented. We will provide you, personally, with the results of any tests performed on you/your child, and would be happy to answer your questions about the study.

Judit E. G. Smits (DVM, MVetSc., PhD)
Professor, Wildlife & Environmental Toxicology
Department of Ecosystem & Public Health,
Faculty of Veterinary Medicine, Univ. of Calgary
3280 Hospital Drive, NW, Calgary, AB, Canada T2N 4N1
Email: jegsmits@ucalgary.ca

We will carefully preserve your/your children's samples (blood, urine, stool and hair) for future use if needed for the next 5 years. We may use the samples for some measurements e.g. toxic metals and their effects on health status. In that case we will maintain the confidentiality about your/your child's identity.

IF I SUFFER A RESEARCH-RELATED INJURY, WILL I BE COMPENSATED?

In the event that you suffer injury as a result of participating in this research, no compensation will be provided to you by Grand Challenges Canada, GIFS, the University of Calgary, Alberta Health Services or the Researchers. You still have all your legal rights. Nothing said in this consent form alters your right to seek damages.

SIGNATURES

Your signature on this form indicates that you have understood to your satisfaction the information regarding your participation in the research project and agree to participate as a participant. In no way does this waive your legal rights nor release the investigators or involved institutions from their legal and professional responsibilities. You are free to withdraw from the study at any time without jeopardizing your health care. If you have further questions concerning matters related to this research, please contact:

Dr. Raqib at: 982706

Or

Dr. Smits +1403-210-7407

If you have any questions concerning your rights as a possible participant in this research, please contact the Chair, Conjoint Health Research Ethics Board, University of Calgary at 403-220-7990 or Bangladesh: **Director,** Bangladesh Medical Research Council (BMRC), Phone: +880 2 8811395, 8828396, 8819311(PABX), Email: info@bmrcbd.org, bmrc@citechco.net

Judit E. G. Smits (DVM, MVetSc., PhD)
Professor, Wildlife & Environmental Toxicology
Department of Ecosystem & Public Health,
Faculty of Veterinary Medicine, Univ. of Calgary
3280 Hospital Drive, NW, Calgary, AB, Canada T2N 4N1
Email: jegsmits@ucalgary.ca

If you have any concerns about the way you've been treated as a participant, please contact an Ethics Resource Officer: Mr. M. A. Salam Khan, Phone No: 9886498 or PABX 8860523-32 Extension 3206

Participant's Name	Signature and Date
Investigator/Delegate's Name	Signature and Date
Witness' Name	Signature and Date

The University of Calgary Conjoint Health Research Ethics Board has approved this research study.

A signed copy of this consent form has been given to you to keep for your records and reference.
