# Supplementary material for: A high-selenium lentil dietary intervention in Bangladesh to counteract arsenic toxicity: study protocol for a randomized controlled trial
Source: Trials. 2016 Apr 27;17:218. doi: 10.1186/s13063-016-1344-y (PMC4848822; doi:10.1186/s13063-016-1344-y)
Supplement: Additional file 3: — Child assent form. (PDF 88 kb) [file 13063_2016_1344_MOESM3_ESM.pdf]

Child Assent Form

Project Title: Mitigating arsenic related health problems in Bangladesh by introducing high-selenium lentils into the everyday diet.

Principal Investigator: Dr. Judit E.G. Smits, Faculty of Veterinary Medicine, Dept. of Ecosystem and Public Health, Phone: 403 210 7407, email: judit.smits@ucalgary.ca
Co-I Dr. Rubhana Raqib, icddr,b, Dhaka, Bangladesh, Phone: +8802-9827068

What is a research study?

- A research study is a way to find out new information about something. Children do not need to be in a research study if they don't want to.

Why are you being asked to be part of this research study?

- In order to stay healthy, it is very important that people eat foods that contain vitamins and so-called micro-nutrients, that help the body to fight illness and poisonous substances. One of these important micronutrients is called selenium. We found out that lentils that grow in a certain region of Canada have a lot of selenium in them. The lentils take up the selenium from the soil.
- You are being asked to take part in this research study because we want to know if these lentils can help people like you stay healthier. About 200 children will be in this study.

If you join the study what will happen to you?

We want to tell you about some things that will happen to you if you are in this study.

- You will be in the study for 6 months (half a year).
- You and your family members will eat the special lentils as dahl, about 65 grams per person.
- We will ask you some questions about your health every two weeks at your home.
- We will ask you about how much lentils you have been eating every day.
- You and your family will come to the Field Office 3 times during the study:
- A health professional will check your health 2 times.
- We will take a small hair sample from the back of your head 3 times.
- We will ask you to give a urine and stool sample 3 times.
- We will use a needle to take some blood from your arm 3 times.
- We will measure your weight 3 times.
- We check the health of your lungs 3 times. For that you will blow into a little machine, which will give us information about your breath.
- You may need to wait for up to 2 hours at the Field Office, but we will provide snacks and drinks for you and your family.

Will any part of the study hurt?

If you agree to give some blood, we will use a needle to take blood from your arm. The needle might hurt a little, but taking the blood will only take half a minute and might be uncomfortable, but this feeling will stop immediately afterwards. There may be some mild bruising, which should go away in a few days.

All other check-ups, like the lung test, will not cause any pain.

If you feel ill or get injured during the trial, tell your parents or the female village health worker that will visit your house, so they can get medical help.

Will the study help others?

- If we find out that the lentils from Canada help people to stay healthier, we will try to grow the same kind of lentils in your country, so that in the future, children like you can eat Bangladeshi lentils and live healthier.

Do your parents know about this study?

- We will talk to your parents about your participation in this study as well. You can talk this over with them before you decide.

Who will see the information collected about you?

- The information collected about you during this study will be kept safely locked up. Nobody will know it except the people doing the research.
- The study information about you will not be given to your parents or teachers. The researchers will not tell your friends or anyone else.

What do you get for being in the study?

- You and your parents will get free lentils from the researchers that you will eat as dahl, just like you are used to.
- You and your parents will get drinks and snacks at the Field Office, while you wait for the medical check-up and sample collection.

Do you have to be in the study?

- You do not have to be in the study. No one will be upset if you don't want to do this study. If you don't want to be in this study, you just have to tell us. It's up to you.
- You can also take more time to think about being in the study.
- If you do not want to give a certain sample at any time, for example blood, you do not have to. You can still participate in the research and give the other samples.

What if you have any questions?

- You can ask any questions that you may have about the study. If you have a question later that you didn't think of now, either you can talk to the village health care worker, or to Dr Raqib, or ask your parents to call Dr. Rubhana Raqib at 9827068.
- You can also take more time to think about being in the study and talk some more with your parents about being in the study.

What choices do you have if you say no to this study?

- If you don't want to be in the study, just say no at the beginning. You will not be able to join the study later. There will be no other opportunity to help with the research.

Other information about the study.

- If you decide to be in the study, please write your name below.
- You can change your mind and stop being part of the study at any time. All you have to do is tell the person in charge. It's okay. The researchers and your parents won't be upset.
- If you wish to stop being part of the study, we will ask you, if we can use the samples you already gave to us, or if you wish that they be destroyed.
- You will be given a copy of this paper to keep.

Would you like to take part in this study?

_____ Yes, I will be in this research study.   _____ No, I don't want to do this.

____________________	____________________	__________
Child's name	signature of the child	Date
____________________	____________________	__________
Person obtaining assent	signature	Date

This research study was approved on ethical grounds by the University of Calgary Conjoint Health Research Ethics Board, the University of Saskatchewan

Biomedical Research Ethics Board and the icddr.b Research Review Committee and Ethics Review Committee.
